# Temperatures of the Mouthpiece of the Bit of Carriage Horses over a Period of 11 Months

**DOI:** 10.3390/ani15172623

**Published:** 2025-09-07

**Authors:** Carina Krcal, Theresia Licka

**Affiliations:** 1 Equine University Clinic, Large Animal Surgery, University of Veterinary Medicine Vienna, Veterinaerplatz 1, 1210 Vienna, Austria; carina.krcal@vetmeduni.ac.at; 2 Department of Veterinary Clinical Studies, Royal (Dick) School of Veterinary Studies, University of Edinburgh, Edinburgh EH 259 RG, UK

**Keywords:** welfare, meteorological conditions, infrared thermography, bit material, bit shape

## Abstract

Besides oral temperature, meteorological parameters are expected to be relevant for mouthpiece temperature, potentially influenced by the material, surface area and weight of the bit. This study measured the temperature of the mouthpieces at the corner of the mouth while they were in use on 58 carriage horses during the four 2024 seasons. Stainless steel, copper, and copper–steel bits were tested in three shapes: Butterfly Liverpool, Liverpool, and Loose Ring Snaffle with four rings. Additionally, surface temperatures of inner thighs, the ground, and buildings were measured using infrared thermography as well as meteorological parameters. Mouthpieces of copper bits reached the highest median temperatures of all materials over all months (31.9 °C) and in August (34.5 °C). In February, the air temperature (median 12.45 °C) was cooler than mouthpiece temperatures (steel median 21.8 °C, copper median 26.4 °C). Mouthpieces of copper–steel Liverpool bits were significantly warmer than those of steel Liverpool bits (+1.1 °C, *p* < 0.026) at wet bulb globe temperatures > 25 °C. Maximum mouthpiece temperatures of steel Butterfly Liverpool bits showed a weaker correlation (Spearman Rho 0.8) with wet bulb globe temperature categories than both steel Liverpool bits and steel Loose Ring Snaffle bits, which demonstrated a full correlation (Spearman Rho 1). Concerning mouthpiece temperatures, bit material, shape, and weather conditions should be considered when selecting bits to ascertain impacts on equine welfare.

## Simple Summary

Horses may experience mouthpiece temperatures as disagreeable or painful sensations in hot or cold outside temperatures. In use, most of the mouthpiece of the bit is in the mouth, with a small part visible at the corners of the mouth. Little is known about the temperature of mouthpieces during use, and this may be affected by bit material, weight, and shape. This study measured the temperature of the mouthpieces at the corner of the mouth while in use on 58 carriage horses during January–November of 2024 in Vienna, Austria. Stainless steel, copper, and copper-steel bits were tested in three common shapes: Butterfly Liverpool, Liverpool, and Loose Ring Snaffle with four rings. Copper bits showed the highest mouthpiece temperatures (median 31.9 °C) over all months. In colder months, mouthpiece temperatures were warmer than the air; for example, in February, the median air temperature was 12.45 °C, while mouthpiece temperatures of steel bits were a median of 21.8 °C and of copper bits a median of 26.4 °C. Mouthpieces of copper–steel Liverpool bits were significantly warmer than those of steel Liverpool bits (+1.1 °C, *p* < 0.026) at wet bulb globe temperatures above 25 °C. The study shows that bit material and weather conditions affect mouthpiece temperature, which is potentially important for horse comfort and welfare.

## 1. Introduction

Effective communication between horse and rider or driver is essential in equestrian activities and has traditionally been facilitated using a bit—comprising a mouthpiece and various components outside of the mouth—a practice that dates back approximately 6000 years [1]. In equine sports, regulatory bodies now require evidence-based guidelines for permissible bit shapes [2,3]. This study addresses this need by investigating mouthpiece temperatures (MT), thereby adding to the scientific basis for informed bit selection and regulation.

Bits vary widely in size, shape, and mechanics [4], with selection typically guided by efficacy and the horse’s sensitivity and training level. Radiographic studies have mapped oral structures with the highest mouthpiece contact [5]. Anatomical features, such as bar length, mandible width, palate shape, and tongue size vary between individuals and determine comfortable mouthpiece fit [5,6]. Critically, mouthpieces can induce pain by stimulating receptors in the interdental space, tongue, buccal mucosa, lips, and mouth commissures, with behavioural cues (e.g., mouth movements, head positions, and facial expressions) potentially signalling discomfort [7]. Moreover, the mouthpiece’s proximity to the mental foramen and mandibular nerve poses the risk of triggering pain responses like head shaking [8], underscoring the welfare relevance of bit design and fit.

In Ireland, most bits are manufactured from stainless steel, with copper as the second most common material; stainless steel mouthpieces are considerably harder than copper [9]. Depending on the country, other materials such as titanium, plastic, and rubber bits are also commonly used. Mouthpiece material has shown no difference in the horse’s salivary pH through oxidation when using titanium and stainless steel bits [10]; excessive salivation has been investigated to be a behaviour not caused by bit-induced pain but rather by other physiological effects such as a reflex response to an oral foreign body [11]. Yet no published analyses exist examining the effects of different mouthpiece materials on equine well-being.

Little is known about the temperatures of the mouthpiece during use. Air temperature and solar radiation are expected to influence the exposed part of the bits (e.g., shanks, rings) directly and the MT indirectly, with effects likely varying according to the material, surface area, and mass of the bit and of the mouthpiece.

**Figure 1:**
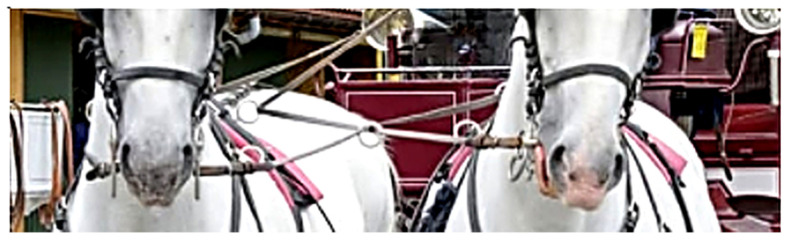
Frontal view of two carriage horses in a team. In the horse on the left side of the image, both shanks and the external parts of the bit are in a relaxed position, with no rein tension applied. In the horse on the right side of the image, unilateral rein tension is visible, causing the bit to shift—moving the mouthpiece on one side into the oral cavity and on the opposite side outward. Photo by the authors.

Solar radiation can increase the temperature of stainless steel roof panels by up to 39.4 °C above air temperature, reaching around 70 °C [12]. Even lower temperatures can potentially damage tissues and compromise equine welfare. While burn injuries from hot sun-exposed surfaces are well-documented in humans [13], equine oral temperature sensitivity remains unclear. With rising summer temperatures and solar radiation exposure, heated bit components pose potential welfare concerns. While skin contact with heated shanks or rings risks thermal injuries, the primary concern is the temperature of the mouthpiece, which is largely within the oral cavity. In a metal mouthpiece, the temperature is expected to be uniform throughout, that is both inside the horse’s mouth and in the small part visible at the corner of the mouth. As unilateral rein tension can pull the side of the mouthpiece into the oral cavity (Figure 1), no part of the mouthpiece that remains always outside the mouth can be defined.

In sports horses, non-contact infrared thermography (IRT) is used to measure skin temperatures [14]. This surface temperature measurement is valuable for monitoring physiological status, welfare, and stress responses [15], provided the animal is suitably prepared and instruments are calibrated. It enables practical health monitoring and injury diagnostics, particularly for hooves, tendons, and muscles [16]. This technology can detect musculoskeletal changes from training adaptation [17] and serves as a non-invasive physiological stress assessment tool [18]. Additionally, IRT measurements at selected surface locations, such as the eye, may serve as indices of body temperature [19].

Wet bulb globe temperature (WBGT) is a useful measure to document the full effect of solar irradiation, humidity, and air temperature. Like Marlin et al., the need to measure WBGT, next to other meteorological measurements of like relative humidity (RH), and air temperature in this study is given to show the effect on equine thermoregulation [20,21].

The present study is part of a large observational investigation into the welfare of carriage horses used in the city of Vienna. Specifically, it aims to describe the relationship between the temperature of the part of the mouthpiece visible at the corner of the mouth and key factors such as bit shape and material, as well as meteorological parameters and surface temperatures of the buildings facing the street at the carriage stands closest to the horses and of the ground below the horse, as well as of the horse’s inner thigh. We hypothesize that the effects of air temperature and solar radiation transferred by the parts of the bit exposed to the elements, i.e., shanks, rings, and the curb chain, will have an impact on the mouthpiece of the bit. Higher air temperatures and increased solar radiation in the warmer months will raise the mouthpiece temperatures of the bit. The results are intended to inform the selection of bits for horses that work outdoors.

## 2. Materials and Methods

### 2.1. Ethical Approval

The study protocol was approved by the Ethics and Animal Welfare committee of the University of Veterinary Medicine, Vienna in accordance with the university’s guidelines for good scientific practice (ETK-160/10/23, 23 November 2023).

### 2.2. Animals

This study included all 58 horses used for carriage driving in Vienna under legal regulations [22], owned by the two largest commercial carriage companies. Horses worked in consistent teams of two, completing tours mainly at a walk, with some trot, lasting 20–360 min per day. Data was collected at designated carriage stands (Michaelerplatz, Albertina, and Stephansplatz) while horses waited in full harness. If rugs were used at the discretion of the driver, they were removed 10 min prior to data collection [23]. Due to the observational nature of the study accompanying normal work routines, the 10 min adaptation period after rug removal could not be extended. Only horses that were used for carriage driving in the city within the rules and legal regulations [22] were included. The veterinary care of the horses remained the responsibility of the owners together with their veterinarians, and vaccinations, dewormings, and oral and other examinations and subsequent treatments were carried out in the routine horse husbandry. The data collection team of the present study was not involved in these measures.

### 2.3. Data Collection

**Figure 2:**
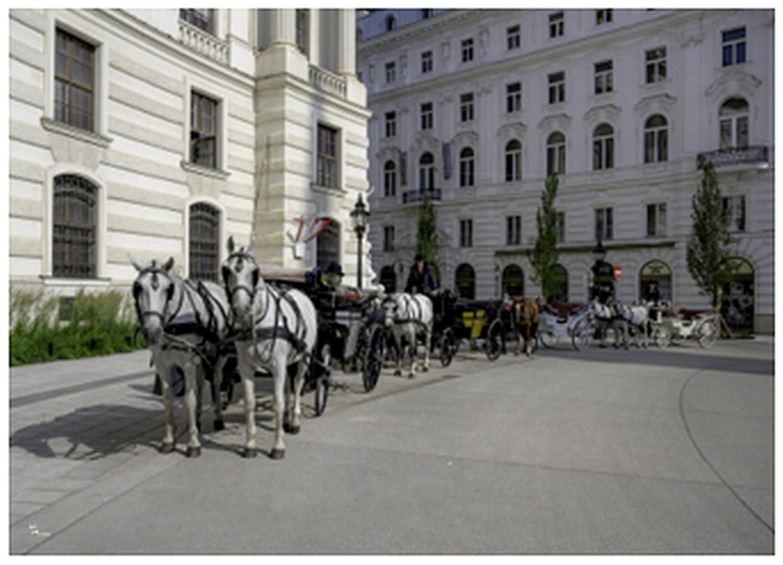
Carriage stands at the location Michaelerplatz, Vienna, Austria. Photo by Hermann Hofer.

Data collection for this study ran from January to November 2024 conducted by six third- to sixth-year veterinary medicine students who had passed the clinical skills examination and were trained, deemed competent, and supervised by an experienced veterinarian. Recorded details included the team’s position in the queue at the carriage stand (Figure 2), bit material, and bit shape. Mouthpiece temperatures were collected when the horses were at stance at the carriage stand. The duration of the preceding tour, the total number of preceding tours, and the time of the examination, as well as the number of minutes that had already been spent at the carriage stand, were documented as the information given by the respective carriage driver. Assessments were carried out over three-hour periods, starting one hour before the forecasted maximum daily temperature [24] at the nearest meteorological station (“Operngasse”). According to legal regulations [22], carriage operations in the city are permitted until air temperatures at that meteorological station reach 35 °C; at this threshold, rides are suspended and animals must be returned to stables.

In total, 693 measurements of MT were obtained over 11 months (see Table 1).

#### 2.3.1. Bit Shape and Material

**Figure 3:**
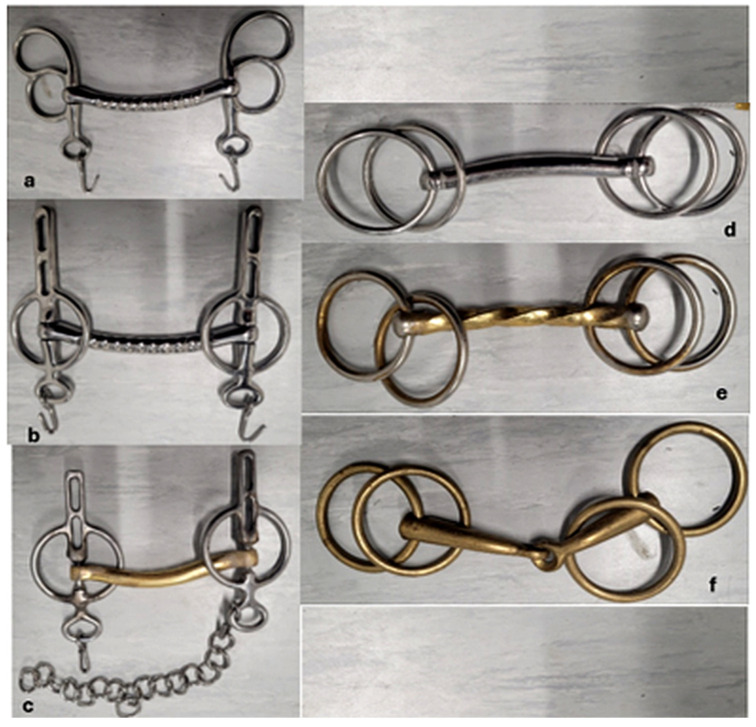
Different bit configurations: (**a**) Butterfly Liverpool in steel; (**b**) Liverpool bit in steel; (**c**) Liverpool bit in copper–steel with the curb chain used for (**a**–**c**); (**d**) Loose Ring Snaffle with 4 rings in steel; (**e**) Loose Ring Snaffle with 4 rings in copper–steel; and (**f**) Loose Ring Snaffle with 4 rings in copper. Photo by the authors.

For the remainder of this paper, bits are defined by bit shape (BS) and bit material (BM); the combination of these is referred to as bit configuration (BC). Three different BSs were in use during the study (Figure 3). The Butterfly Liverpool bit consists of a rigid mouthpiece with shanks and rings, while the Liverpool bit also features a rigid mouthpiece with two straight, long shanks. With both, a curb chain is used that passes under the horse’s chin. The third shape, the Loose Ring Snaffle with four rings, has a rigid or jointed mouthpiece with two loose rings on each side. Three BMs were in use: bits made of polished stainless steel (referred to as “steel”), bits made of polished copper (referred to as “copper”), and bits combining a polished copper mouthpiece with shanks and rings made of polished stainless steel (referred to as “copper-steel”) (Table 2).

#### 2.3.2. Physical Measurements

##### Measurement of the Bits

The size of the bits [mm] was determined measuring the inner surface of the rings and the length of the shanks where present. Bits (n = 93) were weighed [g] using a set of household scales (Crofton, Model 96650, Sattledt, Austria), with the mouthpiece weighed separately by manually lifting rings and or shanks. The total weight of the bit was determined with the chain, if present. The measurements of the size and weight of the bits were made on rest days when bits were not in use, and each bit was measured only once.

**Figure 4:**
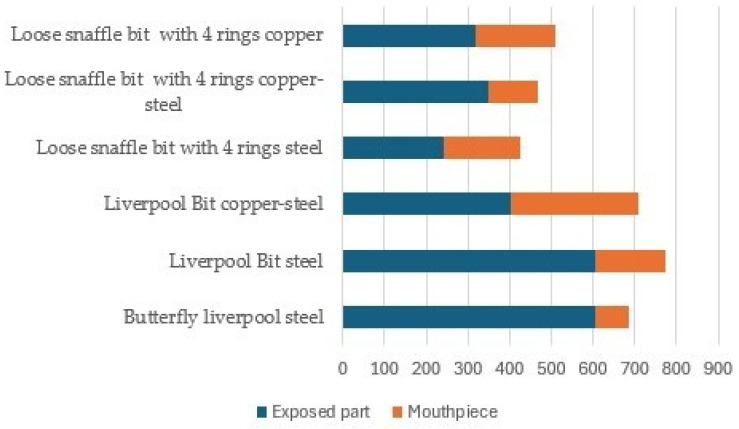
Weights in grams of the mouthpiece (orange) and of the parts of the bit exposed to the elements (shanks and/or rings and curb chain, where present) (blue) are shown.

The relevance of exposure to air temperatures required further definition of permanently exposed parts of the bit, i.e., shanks, rings, and the curb chain versus mouthpiece of the bit, which is largely enclosed in the oral cavity (Figure 4). 

The MT was measured using the surface thermometer “testo 905-T2 (Testo Austria, Vienna, Austria)” The spring-loaded thermocouple band was held in contact with the part of the mouthpiece visible at the corner of the mouth for 10 s before taking the reading. The mouthpiece of the bit was located in the horse’s mouth during the measurement.

##### Surface Temperature of the Horse’s Inner Thigh

**Figure 5:**
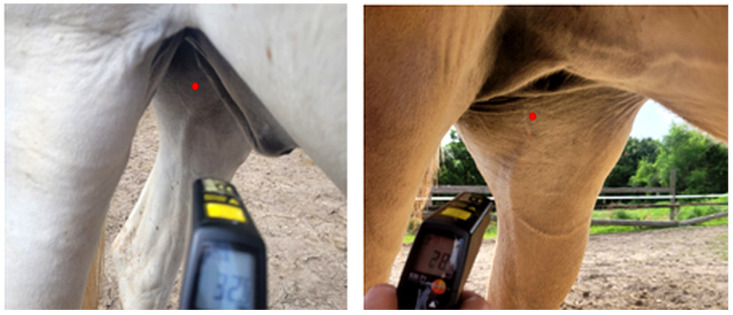
Examples of taking the surface temperature of the horse’s inner thigh of a gelding (**left** image) and a mare (**right** image) using the Testo 830-T1. The Laser pointer marks the position of the femoral vein proximal in the thigh. Photo by the authors.

The surface temperature of the inner thigh (STT) was measured, as rectal body temperature could not be obtained in this observational study. Two non-contact IRT thermometers were used in the study. The Laserliner ThermoSpotPocket (Umabrex, Arnsberg, Germany) (used January–April) was replaced with the Testo 830-T1(Testo Austria, Vienna, Austria) (used April–November) because the former could not save data, necessitating simultaneous manual documentation. All measurements followed a standardized protocol: they were taken at a right angle to the horse’s long axis, approximately one metre from the horse’s side, aiming at a point a hand’s width below the udder (in mares) or prepuce (in geldings) at the location of the femoral canal. This location was chosen to minimize external radiation interference and because the femoral vein was clearly visible (Figure 5).

##### Infrared Thermography

**Figure 6:**
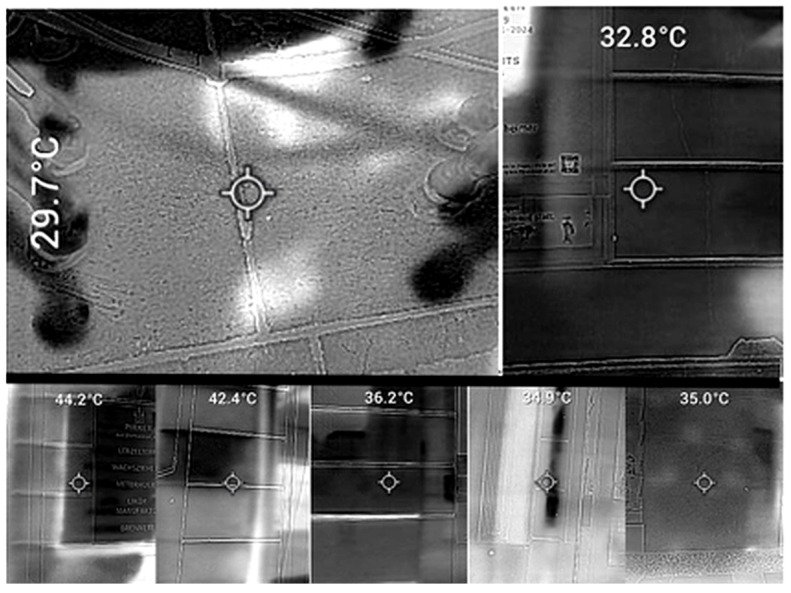
Grayscale set of a thermal image of the ground temperature, top left, showing the ground beneath the horse clearly identified by all four distal limbs. Top right—thermal image of a façade. At the bottom there is a panorama series of images of building surfaces in the sun and in the shade. Temperature values in the images refer to the temperature of the circle in the centre of the image. Photo by the authors.

**Figure 7:**
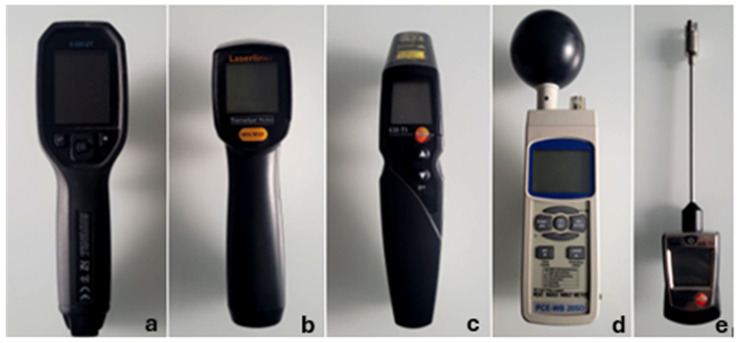
The measurement devices used in the present study. (**a**) FLIR TG165-X MSX (FLIR, Täby, Sweden) Thermal Camera for infrared thermography, (**b**) Laserliner ThermoSpotPocket thermometer (Umabrex, Arnsberg, Germany), (used for infrared thermography, (**c**) Testo: 830-T1 thermometer(Testo Austria, Vienna, Austria) used for infrared thermography, (**d**) heat index WBGT metre PCE-WB 20 SD (PCE, Meschede, Germany) used for meteorological measurements, and (**e**) surface thermometer Testo 905-T2 (Testo Austria, Vienna, Austria) used for measuring the temperature of the mouthpiece. Photo by the authors.

The thermographic measurements of the ground and buildings were carried out using the FLIR TG165-X MSX Thermal Camera (FLIR, Täby, Sweden), (Figure 6 and Figure 7). For the ground temperature (GT), measurements were taken of the ground below the horse, with the camera placed approximately one metre beside either the left or right side of the horse and angled downward at 45°. At least one front leg and one hind leg were included in the image. For the Specific Facade Temperature (SFT), the temperature of the surface of the building closest to the animal was measured at a height of about one metre. During August to November, the Panorama Façade Temperature (PFT) was additionally documented as follows: the surface temperature of buildings facing the street at the carriage stands (Michaelerplatz, Stephansplatz, and Albertina) was recorded by measuring each visible building surface from both the first position of the carriage stand and one waiting position. Temperatures for shaded façades and sunlit façades were documented. Figure 6 illustrates a portion of the building surfaces captured in the panorama façade view. The specific measurement points on the façades were determined as described for the SFT.

All the measurements of GT, SFT, and PFT were obtained within the same hour of MT.

An emissivity value of 0.88 was used for the measurement of these surface temperatures [25,26]. A grayscale thermal image was obtained, with the highest temperature represented as white and the coolest as black. The laser pointer was used to centre the image around the area of interest, and only the values of the temperature of the area of interest was taken forward (Figure 6).

##### Meteorological Measurements

A heat index wet bulb globe temperature (WBGT) thermometer metre PCE-WB 20 SD (PCE, Meschede, Germany), (Figure 7) on a tripod was used for the meteorological measurements of WBGT [°C], relative humidity (RH) [%], and air temperature (TA) [°C].

The tripod was placed on the sidewalk one to three metres away from the horse at the height of about one metre. The meteorological data collected were assigned to both horses of the team. The WBGT device (PCE, Meschede, Germany) requires a 10 min stabilization period to provide reliable measurements, and readings were therefore recorded at 10 min intervals. Meteorological data were matched to the time each horse was examined.

### 2.4. Data Processing

#### 2.4.1. Instrument Cross Validation of the Infrared Thermometers

From 1 March to 15 April, both thermometers were used concurrently; for validation, each horse’s temperature was recorded three times using device 1 (Laserliner ThermoSpotPocket, Umabrex, Arnsberg, Germany) and once using the device 2 (Testo: 830-T1, Testo Austria, Vienna, Austria) (Table 3).

A linear regression (R^2^ = 0.9976) was calculated between the mean of three measurements of device 1 and the corresponding measurement of device 2. Based on this, results of device 1 were recalculated using the regression equation to obtain comparable results.

°C_Testo = °C_Laserliner × 0.66 + 9.99

Thus, results of devices 1 and 2 were highly significantly correlated (PCC = 0.734; *p* < 0.01).

#### 2.4.2. Surface Area of Different Bit Configurations

**Figure 8:**
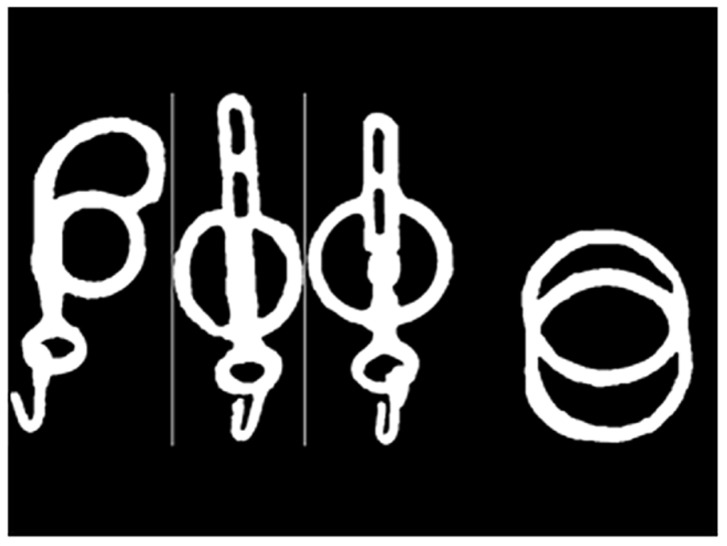
Binary images of the left-sided parts of the bit exposed to sunlight in the different bit configurations. From **left** to **right**: Butterfly Liverpool, Liverpool bit steel, Liverpool bit copper–steel, and Loose Ringe Snaffle with 4 rings. Photo by the authors.

Lateral images of the left-sided parts of the BC that are directly exposed to sunlight were converted to grayscale (8-bit) and scaled using length measurements (Table 3); then, thresholding (Binary Contrast Enhancement) was used (Figure 8) to determine the 2D surface area using histograms of black and white pixels in Image J (Version 1.54g) (Table 4).

### 2.5. Statistical Analysis

Data were analyzed using JASP Team (2024) JASP (Version 0.19.3).

After identifying the nonparametric distribution using the Shapiro–Wilk test, Kruskal–Wallis tests were used to identify differences in MT between BM, BS, and BC. Dunn’s post hoc comparisons were used to determine the sources of differences between bits for the whole data collection period (January–November 2024) and separately for each single month.

Four groups of WBGT (<10°, 10–20°, 20–25°, and >25°) were formed to document differences between the BC using Friedman’s test followed by Conover’s post hoc test.

Post hoc significance values were adjusted using the Bonferroni correction for multiple tests. For all data analyses, the statistical significance was set at *p* < 0.05.

Spearman’s rank correlations were used to identify the relationship between GT, FT, STT, MT, BM, WBGT, RH, TA, surface area of the exposed part of the bit, total weight of the bit, weight of the external part of the bit, and weight of the mouthpiece.

Variables were categorized to their thermal conductivity for bit material (lowest to highest): steel with a thermal conductivity around 14–16 W/m·K as 1, copper–steel as 2, and copper with a thermal conductivity approximately 399–401 W/m·K as 3; and to their surface area of the exposed area of the bit for bit shape (highest to lowest): Liverpool with 36,099.2–29,147 mm^2^ as 1, Butterfly Liverpool with 20,349.6 mm^2^ as 2, and Loose Ring Snaffle with 14,280.6 mm^2^ as 3. Correlations were calculated for all parameters over the whole data collection period (January–November 2024), as well as for each single month. Spearman’s rank correlations were reported as r_s_(n) and *p*.

## 3. Results

### 3.1. Animals

A total of 58 horses were used for this study. Data collection began with 51 horses. During the study period, seven additional horses were included, while four horses were excluded due to health issues. Also, one horse was excluded because it lost its team partner.

Horses worked in established teams of two, but team partners were changed for three individuals over the course of the study.

### 3.2. Meteorological Measurements over the Year

The study started in the second warmest winter in recorded history in Austria, followed by the warmest spring and summer. A new record of 52 days of heat (average AT over 24 h of ≥30 °C) was recorded in 2024 in the centre of Vienna [27]. The meteorological measurements obtained at the data collections for every team of horse are presented in Table 5.

### 3.3. Comparison of Bits

To select the appropriate BS for horses that work in different weather conditions, the effect on the MT of the different BSs were compared. The Loose Ring Snaffle with four rings had the highest temperature over the 11 months, with a median value of 31.5 °C (min: 14.9 °C; max: 36.8 °C), being greater by 2.6 °C than the Liverpool bit (median 28.9 °C, min: 15.5 °C; and max: 37 °C) and 2.5 °C more than the Butterfly Liverpool bit (median 29; min: 13.8; and max: 36.6). See also the Supplementary Figure S2. Significant differences were found for all 11 months between the Butterfly Liverpool bit and Loose Ring Snaffle with four rings (*p* < 0.001), for April between the Butterfly Liverpool and Loose Ring Snaffle with four rings (*p* < 0.001), and between the Liverpool bit and Loose Ring Snaffle with four rings (*p* < 0.01). No statistically significant differences were found for the other months (*p* > 0.05). See also the Supplementary Table S4.

MT can differ due to the thermal conductivity of different BMs. To help with selecting bits to ascertain impacts on equine welfare, BMs were compared. Over all data collections, copper was identified as the warmest bit (31.9 ± 19.8), being 2.4 °C higher than steel (29.5 ± 23.2) and 4 °C higher than copper–steel (27.9 ± 14.8). See also the Supplementary Figure S1. Over 11 months, a significant difference in the MT between steel and copper bits (*p* < 0.002) was found. The Loose Ring Snaffle with four rings had the highest temperature over the 11 months, with a median value of 31.5 °C (min: 14.9 °C; max: 36.8 °C), being greater by 2.6 °C than the Liverpool bit (median 28.9 °C, min: 15.5 °C; and max: 37 °C) and 2.5 °C than the Butterfly Liverpool bit (median 29. min: 13.8; and max: 36.6). For February, significant differences were determined between steel and copper bits (*p* < 0.044) and between steel and copper–steel bits (*p* < 0.004); for March, between steel and copper bits (*p* < 0.013); for April, between steel and copper bits (*p* < 0.001); and for May, between steel and copper bits (*p* < 0.001). No statistically significant difference was found for the other months (*p* > 0.05). See also the Supplementary Table S3.

**Figure 9:**
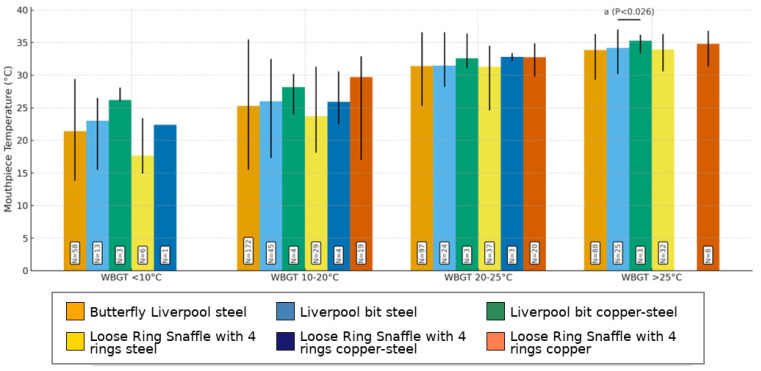
Median values (min–max) of mouthpiece temperatures [°C] of the bit configurations in the groups of WBGTs. Four groups of WBGTs (<10°, 10–20°, 20–25°, and >25°) were formed to document differences between bit configurations using Friedman’s test, followed by Conover’s post hoc test. Post hoc significance values were adjusted using the Bonferroni correction for multiple tests. Superscript a indicates a significant difference between the Liverpool bit steel and the Liverpool bit copper–steel in the category WBGT > 25 °C (*p* < 0.026).

For bit configurations, a significant difference was found in the category of WBGT > 25 °C between the Liverpool Bit in steel and Liverpool bit in copper–steel (*p* < 0.026. Figure 9). No statistically significant difference was found in the other groups (*p* > 0.05, Figure 9). See also the Supplementary Tables S5 and S6.

### 3.4. Differences Between Mouthpiece Temperature, Meteorological Measurements, and Surface Temperatures

To identify a comparable value to the MT, the differences between MT, meteorological measurements, and surface temperatures were demonstrated. Therefore, carriage drivers can estimate with comparison to other values if the MT can be uncomfortable for their horses. The differences between MT and TA, RH, WBGT, GT, SFT, and STT were calculated for every data collection day, and the mean values (min–max) are shown in Table 6 The smallest difference was found between the MT and STT over all months, but for the single months of April, September, October, and November, the difference between the MT and GT is the smallest (Table 6).

### 3.5. Correlations Between Mouthpiece Temperature, Meteorological Measurements, and Surface Temperatures

Significant Spearman’s rank correlations are shown in Table 7, based on all parameters of all individual measurements available for all months. The mouthpiece temperature of the bit and meteorological measurements and surface temperatures are presented to compare the correlation and connection (Table 7).

The information on façade temperatures was only available for the months of August-November, and in Table 8, daily median values of the façade in the sun and in the shade are given together with daily maxima and daily median values of all the measurements obtained within the same hour for the MT and GT. Spearman’s rank correlations between these MT values and the GT and façade values of the same day are also given. It demonstrates the coldest and warmest month and shows the effect of the surroundings (ground temperature, façade temperature) on the mouthpiece of the bit (Table 8). See also the Supplementary Table S1.

## 4. Discussion

This study investigated the MT across various BSs and BMs in carriage horses as an observational study working in Vienna over an 11-month period in 2024.

To the authors’ knowledge, the temperatures of mouthpieces have not yet been documented during use. Previously, titanium and stainless steel MTs were documented immediately after use in horses that had worked athletically for 30 min in a crossover design study carried out in December in northern Italy [10]. Their ambient maximum temperatures ranged between 9 °C and 11 °C, according to available meteorological information [28], close to the air temperatures of January and February in the present study. Both titanium bits (mean 30.23 °C) and stainless steel bits (mean 32.10 °C) were considerably warmer than the mouthpieces measured in the present study in these two months (median values: January 21.5 °C, February 22.2 °C). This discrepancy is clearly attributable to the low level of exercise in the carriage horses investigated in the present study.

The maximum temperatures documented for mouthpieces in the present study are all below the documented oral temperatures in horses [29]. In human medicine, a review investigated whether oral temperature correlates reliably with the core body temperature, but relevant differences were found both at rest and during exercise, probably due to TA and saliva [30]. Similar results were found for manatees [31]. In dogs, the temperature at the gingiva was found to be suitable for detecting hyperthermia, but it did not reliably indicate the core body temperature [32]. In horses, the oral buccal mucosa temperature, measured using infrared thermometers, was significantly higher than the rectal temperature obtained using both infrared (+0.15 °C) and mercury (+0.18 °C) thermometers [29]. Thus, the maximum temperatures of the mouthpiece obtained cannot be regarded as detrimental to the well-being of the horse based on the results of the present study.

The same cannot be said for the lowest temperatures measured at the mouthpieces in the present study, even though data collection was carried out during the warmest hours of each day. In humans, the threshold for thermal pain, experienced as, e.g., “burning” or “pulling”, was investigated for different locations within the mouth [33]. In the human oral cavity, the gingiva was found to be most sensitive to cold and least sensitive to heat. The cold pain threshold was above 20 °C in nearly all participants, while the heat pain threshold was at more than 40 °C in all participants [33]. The mouthpiece of the horse is typically placed on the bars of the mandible, where it is in contact with the mucous membrane [34] and on rein tension in contact with the gingiva at the position of the first premolar tooth. Since lateral margins of the tongue cover the bony ridges of the bars of the mouth, thereby supporting most of the bit-induced pressure, unless the tongue has been retracted or is lying over the bit [9], parallels between the mouth sensitivities of horses and humans may exist. Therefore, we would expect horses to tolerate the maximum MT documented in the present study without discomfort, while some discomfort, or potentially even pain, might be elicited at the minimum MT documented.

Temperature sensitivity varies greatly between individuals. One indicator of temperatures acceptable to horses is the preferred temperature of drinking water, even though water contact with the oral cavity is very short. A study aimed at optimizing hydration has identified the temperature preferences of ponies for drinking water. The optimal water temperature ranged from 7 °C to 18 °C, and in winter, ponies consumed approximately 40% more water warmed to this range compared to near-freezing temperatures. However, paradoxically, when offered a choice, they often preferred colder water despite consuming less of it overall [35]. Based on these results, it is reasonable to expect that bit temperatures measured during colder months may fall within an acceptable range. Nonetheless, the distinction between the brief exposure to cold temperatures during drinking and the continuous exposure associated with a mouthpiece must be considered.

Environmental conditions, comprehensively represented by WBGT, significantly influenced the MT in the present study. Materials of a darker colour absorb more solar radiation, leading to higher temperatures, and the present study observed that bits made of copper reached a higher median MT over the 11 months, even though steel bits reached the highest maximum MT of all bits, with 37 °C in June. Thermal conductivity, i.e., the ability of a material to transfer heat, is very high in copper (approximately 399–401 W/m·K) [36]. This allows the copper bit to quickly equilibrate with the horse’s mouth temperature, which may enhance comfort for the animal. In contrast, steel has a lower thermal conductivity (around 14–16 W/m·K) [37], resulting in slower heat transfer and potentially prolonged temperature differences between the mouthpiece and the mouth. This is the reason for the recommendation to “pre-warm” the mouthpiece before use in colder weather [37].

Only one BS, the Loose Ring Snaffle, was in use in copper, steel, and copper–steel, allowing for direct comparisons of the BM, and in copper–steel, it was warmer than others in winter and cooler than others in summer. Consequently, this combination should be considered for horses working in cold and hot temperatures. The relevance of the BM is further supported by the results of the three BMs independent of the BS.

The area of the bit exposed to solar radiation was expected to correlate with the MT; this was only significant in February, where colder air and some sunshine were present together. Based on the principles of thermal mass, the weight of the bit (divided into the external parts and the mouthpiece) was also expected to correlate with the MT, and this was confirmed as significant for all months. Also, the maximum MT in the heat was measured in the heaviest bit, i.e., the steel Liverpool bit, with about 78% of the weight exposed, while the slightly lighter copper steel Liverpool bit, with about 57% of the weight exposed, was the warmest in the cold season. This shows the relevance of the weight parameters of the bits for MT.

For future studies, temperature sensors could be embedded within mouthpieces to enable monitoring, similar to a study in humans where an intra-oral portable micro-electronic device attached to the teeth measured real-time temperature and humidity in the oral cavity [38]. Interventional studies using mouthpieces cooled or warmed to different temperatures could be used to determine the acceptable temperature ranges of mouthpieces for horses. In the meantime, in practical settings where the direct measurement of mouthpiece temperature is not feasible, GT and SFT may serve as indications of the MT, given their relatively small difference to the MT.

In the present study, several limitations need to be considered. As an observational study, no interventions were implemented to manipulate the MT or assess indicators of negative effects, such as discomfort. They include that MT was measured at the small part visible at the corner of the mouth and not at the centre of the mouthpiece. Potential differences between the temperatures at these locations could not be assessed, although based on the thermal conductivity of the metals of the mouthpieces, this difference is probably small. Additionally, studying a broader variety of BCs would have enabled more generalized conclusions. Since the overall focus of the study was on horses working in hot conditions, data collection was conducted around the time of the highest expected daily temperatures. However, based on our findings, collecting data during the coldest periods of the day may also be relevant to document the minimum MT reached, as cool mouthpieces might elicit discomfort.

## 5. Conclusions

The study shows that bit material and shape are key determinants of mouthpiece temperature, with copper producing higher median temperatures and steel reaching higher maxima. While bit weight had some influence, material properties and environmental conditions were more decisive. Practically, this means that bit choice should consider both the material and shape in relation to the expected climate: in hot conditions, steel bits may reach higher, potentially uncomfortable peak temperatures, whereas in cooler conditions, copper may warm more quickly and be potentially more comfortable. Careful selection tailored to environmental conditions may therefore improve equine comfort and welfare.

## Figures and Tables

**Table 1:** Number of data points (n) of mouthpiece temperatures (temp), bit shapes, and bit materials obtained during each of the months (January–November) and during all months.

2024	Mouthpiece Temp n	Bit Shape n	Bit Material n
January	10	9	9
February	56	56	56
March	42	42	42
April	72	73	73
May	77	79	79
June	111	111	112
July	55	55	55
August	87	88	88
September	75	76	76
October	55	59	59
November	53	55	55
January–November	693	703	704

**Table 2:** Bit shapes and materials used for the 58 carriage horses over the duration of the present study; for some horses more than one bit was used at the discretion of the driver.

Bit Materials	Butterfly Liverpool	Liverpool	Loose Ring Snaffle (4 Rings)
Steel	50	15	14
Copper	-	-	7
Copper–Steel	-	4	3

**Table 3:** Mean values and standard deviation of measurements of surface temperature of the horse’s inner thigh using two different thermometers. The wet bulb globe temperature, relative humidity, and air temperature at the time of these measurements are also given.

	Mean Values	Standard Deviation	N
Infrared Thermometers			
Laserliner ThermoSpotPocket (Device 1)	30.72 °C	1.98 °C	107
Testo: 830-T1 (Device 2)	31.53 °C	2.24 °C	321
Meteorological parameters			
Wet bulb globe temperature	15.26 °C	4.41 °C	107
Relative humidity	47.91%	14.68%	107
Air temperature	19.69 °C	5.95 °C	107

**Table 4:** Measurements of the 2D surface area exposed to sunlight of the left side of the different bit configurations (Butterfly Liverpool, Liverpool bit, and Loose Ring Snaffle with 4 rings) using Image J, based on distances defined by the manufacturer. Doubling the left-sided surface area was performed to calculate the total surface area exposed to sunlight on both sides of the bit.

	Butterfly Liverpool	Liverpool Bit Steel	Liverpool Bit Copper–Steel	Loose Ring Snaffle 4 Rings
defined length of 10 mm [pixel]	2.38	2.38	2.38	2.38
black [pixel]	77,123	14,9838	76,819	42,541
white [pixel]	24,216	42,958	34,685	16,994
left-sided white area [mm^2^]	10,174.8	18,049.6	14,573.5	7140.3
area [mm^2^] of all exposed parts	20,349.6	36,099.2	29,147	14,280.6

**Table 5:** Median value (min–max) of meteorological measurements obtained for each data collection of every team of two horses for each month and for all 11 months (January–November).

2024	Wet Bulb Globe Temperature [°C]	Relative Humidity [%]	Air Temperature [°C]	N
January–November	19.6 (5.5–30.3)	44.3 (27.2–96.9)	26.35 (6.2–39.1)	777
January	6.1 (5.5–11.8)	77 (60.2–96.9)	7.4 (6.2–14.7)	32
February	9.5 (7–12.3)	49.15 (43.9–91.9)	12.45 (7.5–16)	56
March	10.2 (6–15.8)	57.1 (37.1–96.6)	12.7 (7.3–19.9)	42
April	18.5 (13.8–22.9)	39.4 (32–48.3)	24.4 (16.5–30.3)	73
May	19.6 (15.8–25.1)	36.9 (27.2–53.1)	26.4 (20.9–34.5)	79
June	25.1 (19.8–30.3)	39.1 (34.2–56.6)	31.9 (23.8–39.1)	112
July	27.5 (21.7–29.4)	42.9 (31.4–51.3)	33.6 (28.7–38.4)	55
August	25.5 (22.3–29.4)	42.4 (34.4–53.1)	32 (27.4–36.2)	88
September	23 (17–25.7)	45.9 (30–54.7)	28.6 (22.1–34.4)	77
October	14.8 (12.5–16.2)	55.1 (48.6–67.2)	18.3 (15.9–30.3)	61
November	14.5 (7.6–14.5)	61 (48.9–61)	17.6 (10.3–17.6)	55

**Table 6:** Median values (min–max) of differences in mouthpiece temperature (MT) [°C] and surface temperature of the thigh (STT) [°C], air temperature (TA) [°C], wet blub global temperature (WBGT) [°C], ground temperature (GT) [°C], and Specific Façade Temperature (SFT) [°C] for single months and for all months (January-November) obtained during 2024 in the city of Vienna (n = 693).

	MT [°C]	MT-STT [°C]	MT-TA [°C]	MT-WBGT [°C]	MT-GT [°C]	MT-SFT [°C]
All months	29.8 (13.8–37)	2.9 (0–17.2)	3.9 (0–19.9)	8.9 (1.2–21.7)	3.4 (0–19.3)	3.6 (0–18.6)
January	21.5 (17–26)	7.2 (0.6–11.8)	7.7 (5.7–11.3)	10.2 (8.6–14.3)	9.7 (8.2–12.5)	7.5 (4.7–12.7)
February	22.2 (13.8–29.4)	5.7 (0.7–11.8)	10.3 (2.8–11.6)	13.1 (6.7–14.3)	8.5 (2.4–12.5)	7.1 (0.3–10.4)
March	24.5 (15.5–32.5)	6.1 (0.6–17.2)	10.9 (5.7–19.9)	13.1 (8.6–21.7)	7.6 (5.1–19.3)	7.7 (4.7–17.6)
April	29.1 (18.1–32.9)	3.6 (0–12)	5.4 (0.1–12.5)	10.8 (4.3–16.1)	3.4 (0–9.5)	2.3 (0–8.7)
May	29.3 (24.6–32.9)	2.7 (0–7.3)	3.6 (0.1–8.6)	9.6 (4.2–14)	3.2 (0–14.6)	2.0 (0–18.6)
June	32.0 (24.6–37)	1.6 (0–7.6)	2.6 (0–9.2)	8.0 (2.6–15.7)	3.1 (0–14.2)	2.7 (0–12.5)
July	33.3 (29.1–36.4)	1.4 (0–4.9)	2.3 (0.1–7.8)	6.6 (1.2–11.6)	3.7 (0.1–15.7)	5.8 (0.4–12.9)
August	33.1 (28.2–36.2)	1.3 (0–5.2)	2 (0–6.8)	8 (2.8–11.9)	2.3 (0.2–12.4)	5.2 (2.3–8)
September	32.9 (23.1–36.6)	2.5 (0.1–9.4)	2 (0.1–8)	8.6 (3.9–13.6)	1.7 (0–13.6)	4.4 (1.7–7.1)
October	22.2 (15.5–26.6)	8.3 (1.8–14.3)	4.9 (0.2–10.2)	6.8 (2.5–11.8)	1.8 (0.3–7.6)	2.5 (0.8–4.2)
November	22.2 (14.9–32.4)	6.3 (0.1–13.5)	6.5 (2.7–14.8)	9.5 (5.8–17.9)	5.7 (0–14.6)	1.1 (4–1.9)

**Table 7:** Spearman’s rank correlation coefficients (r_s_), *p*-values, and sample sizes (n) for the relationships between all data points of mouthpiece temperature (MT) and bit material (BM), bit shape (BS), relative humidity (RH), air temperature (TA), wet bulb globe temperature (WBGT), surface temperature of the inner thigh (STT), ground temperature (GT), weight of the bit (BW), weight of the shanks, rings, and chain (SRCW), weight of the mouthpiece (MW), and surface area of the shanks and rings (SRA). All significant results are shown; time periods are given as the full data collection period of January to November (all months), as well as separately for the coldest month (February) and the hottest month (July).

Correlation of MT with	February (n = 56)		July (n = 55)		All Months (n = 693)	
	r_s_	*p*-Value	r_s_	*p*-Value	r_s_	*p*-Value
BM					0.139	<0.001
BS					0.152	<0.001
RH	−0.356	<0.007	0.370	<0.005	−0.633	<0.001
TA	0.394	<0.030			0.851	<0.001
WBGT	0.312	<0.019			0.880	<0.001
STT	0.372	<0.005	0.419	<0.001	0.793	<0.001
GT	0.277	<0.039	0.467	<0.001	0.822	<0.001
BW					−0.092	<0.015
SRCW					−0.154	<0.001
MW					0.165	<0.001
SRA	0.373	<0.005				

**Table 8:** For the months of August and November, as well as for the months August-November, median (minimum–maximum) values of the daily median values of the façade in the sun (Fsun) and in the shade (Fsha) are listed together with the daily maximum and daily median mouthpiece temperatures (MT) and ground temperatures (GT), obtained within the same hour. Significant Spearman’s rank correlations with MT values are shown between values with the same superscript as follows: (a) r_s_ = 0.841, *p* < 0.036; (b) r_s_ = 0.890, *p* < 0.001; (c) r_s_ = 0.858, *p* < 0.001; (d) r_s_ = 0.839, *p* < 0.001; (e) r_s_ = 0.892, *p* < 0.001; (f) r_s_ = 0.857, *p* < 0.001; (g) r_s_ = 0.902, *p* < 0.001; (h) r_s_ = 0.792, *p* < 0.001; and (i) r_s_ = 0.916, *p* < 0.001.

Month(s)	Max MT [°C]	Median MT [°C]	Max GT [°C]	Median GT [°C]	Median Fsun [°C]	Median Fsha [°C]
August	35.2 (34.2–36.2)	33.4 ^a^ (31.7–35.2)	39.4 (35.9–45.0)	34.0 ^a^ (31.7–36)	41.5 (39.9–42.8)	35.45 (34.1–37.3)
November	25.15 (17.3–32.4)	20.8 (16.3–23.7)	17.25 (13–25.3)	13.27 (9.2–18.4)	21.7 (17.7–28.0)	15.8 (12.9–21.1)
August–November	32.4 ^b,c,d,e^ (17.3–36.6)	25.7 ^f,g,h,i^ (16.3–35.2)	31.3 ^b,f^ (13–45)	26.4 ^c,g^ (9.2–36.5)	35.3 ^d,h^ (17.7–42.9)	26.0 ^e,i^ (12.9–38.8)

## Data Availability

The original contributions presented in the study are included in the article and Supplementary Materials; further inquiries can be directed to the corresponding author.

## Supplementary Materials

The following supporting information can be downloaded at: https://www.mdpi.com/article/10.3390/ani15172623/s1. Table S1. Sizes as lengths and diameters of all bit configurations used. Table S2. Spearman correlation coefficients (R) and respective *p* values (P) throughout the year (Jan-Nov) and for each month between mouthpiece temperature and bit material, surface temperature of the thigh (STT) [°C], air temperature (TA) [°C], wet blub globe temperature (WBGT) [°C], relative humidity (RH) [%], total weight of the bit [g], weight of the exposed part of the bit, shanks, rings, and chain (weight exposed) [g], weight of the mouthpiece [g], area of the shanks and rings [mm2], and ground temperature (GT) [°C]. Table S3. Median values (min–max) of mouthpiece temperature [°C] between bit material (steel, copper, and copper–steel) over the whole data collection period of January–November 2024 and single months with Kruskal–Wallis test and Dunn-test, while superscripted values within rows are significantly different (*p* < 0.05); N = 695. Table S4. Median values (min–max) of mouthpiece temperature [°C] between bit shapes (Butterfly Liverpool, Liverpool bit, and Loose Ring Snaffle with 4 rings) over the whole data collection period of January-November 2024 and single months with Kruskal–Wallis test and Dunn-test, while superscripted values within rows are significantly different (*p* < 0.05); N = 692. Table S5. Median values (min–max) of mouthpiece temperatures [°C] of the bit configurations in the groups of WBGTs. Four groups of WBGTs (<10°, 10–20°, 20–25°, and >25°) were formed to document differences between bit configurations using Friedman’s test followed by Conover’s post hoc test. Post hoc significance values were adjusted using the Bonferroni correction for multiple tests. Superscript a indicates a significant difference between the Liverpool bit steel and the Liverpool bit copper–steel in the category WBGT > 25 °C (*p* < 0.026). Table S6. Median values (min–max) of mouthpiece temperatures [°C] for the bit configurations (defined by shape and material) over the whole data collection period (all months) and the single months. Figure S1. Mouthpiece temperatures of different bit materials (steel, copper, and copper–steel) over the single months. Temperatures are in °C. Figure S2. Mouthpiece temperatures of different bit shapes over the months; median bit temperatures [°C] of the exposed part of the mouthpieces of different bit shapes (Butterfly Liverpool, Liverpool, and Loose Ring Snaffle with 4 rings) over 11 months.

## Abbreviations

The following abbreviations are used in this manuscript:

BM	Bit material
BS	Bit shape
BC	Bit configuration
TA	Air temperature
RH	Relative Humidity
WBGT	Wet Bulb globe temperature
MT	Mouthpiece temperature
IRT	infrared thermography
N	Number of data points
GT	Ground temperature
SST	Specific façade temperature
PFT	Panorama façade temperature
STT	Surface temperature of the inner thigh
BW	Weight of the bit
SRCW	Weight of the shanks, rings, chain
MW	Weight of the mouthpiece
SRA	Surface area of the shanks and rings
