# Supplementary material for: Temperatures of the Mouthpiece of the Bit of Carriage Horses over a Period of 11 Months
Source: Animals (Basel). 2025 Sep 7;15(17):2623. doi: 10.3390/ani15172623 (PMC12427211; doi:10.3390/ani15172623)
Supplement: Supplementary file 1 [file animals-15-02623-s001.zip › animals-3799505-supplementary.pdf]

Table S1. Sizes as lengths and diameters of all bit configurations used.

Bit Shape and Material(s)	Lengths Shanks [mm]	Diameters [mm]
Butterfly Liverpool steel	Shanks 140	Upper Ring 40<br>Lower Ring 45
Liverpool bit steel	Shanks 175	Ring 60
Liverpool bit copper–steel	Shanks 160	Ring 70
Loose Ring Snaffle with 4 rings steel		Ring 65
Loose Ring Snaffle with 4 rings copper–steel		Ring 65
Loose Ring Snaffle with 4 rings copper		Ring 65

Table S2. Spearman correlation coefficients (R) and respective *p* values (P) throughout the year (Jan-Nov) and for each month between mouthpiece temperature and bit material, surface temperature of the thigh (STT) [°C], air temperature (TA) [°C], wet blub globe temperature (WBGT) [°C], relative humidity (RH) [%], total weight of the bit [g], weight of the exposed part of the bit, shanks, rings, and chain (weight exposed) [g], weight of the mouthpiece [g], area of the shanks and rings [mm2], and ground temperature (GT) [°C].

Variable		Year	February	March	April	May	June	July	August	Sep-tember	October	Novem-ber
Bit material	R	0.139	0.213	0.251	0.369	0.441	0.156	0.024	0.173	0.049	0.128	0.349
	P	0.001	0.115	0.109	0.001	0.001	0.102	0.860	0.110	0.675	0.352	0.010
STT	R	0.793	0.372	0.134	0.338	0.202	0.589	0.419	0.256	0.695	0.358	0.307
	P	0.001	0.005	0.397	0.004	0.078	0.001	0.001	0.017	0.001	0.007	0.025
WBGT	R	0.880	0.276	0.233	0.490	0.480	0.736	0.198	0.452	0.708	0.319	0.543
	P	0.001	0.039	0.137	0.001	0.001	0.001	0.147	0.001	0.001	0.018	0.001
RH	R	−0.633	−0.356	−0.222	−0.270	−0.554	−0.579	0.370	−0.170	−0.699	−0.419	0.507
	P	0.001	0.007	0.158	0.022	0.001	0.001	0.005	0.116	0.001	0.001	0.001
TA	R	0.851	0.312	0.231	0.491	0.547	0.750	0.099	0.453	0.730	0.385	0.546
	P	0.001	0.019	0.141	0.001	0.001	0.001	0.471	0.001	0.001	0.004	0.001
Total bit weight	R	−0.092	0.075	0.011	−0.350	−0.087	0.053	−0.019	0.161	−0.027	−0.036	0.067
	P	0.015	0.583	0.943	0.003	0.450	0.581	0.888	0.135	0.817	0.793	0.635
Weight exposed	R	−0.154	−0.213	−0.235	−0.359	−0.164	−0.003	−0.059	0.031	−0.056	−0.131	0.020
	P	0.001	0.115	0.135	0.002	0.154	0.974	0.666	0.774	0.632	0.339	0.887
Weight mouth-piece	R	0.165	0.103	0.217	0.272	0.228	0.092	0.070	0.109	0.089	0.146	0.034
	P	0.001	0.452	0.168	0.021	0.046	0.338	0.614	0.315	0.446	0.288	0.808
Area shanks and rings	R	−0.015	0.373	0.052	−0.195	0.029	0.269	−0.032	0.132	−0.303	−0.215	−0.150
	P	0.684	0.005	0.743	0.100	0.801	0.004	0.814	0.223	0.008	0.115	0.283
GT	R	0.822	0.277	0.16	0.327	0.147	0.529	0.467	0.46	0.675	0.346	0.489
	P	0.001	0.039	0.238	0.005	0.203	0.001	0.001	0.001	0.001	0.01	0.001

Table S3. Median values (min–max) of mouthpiece temperature [°C] between bit material (steel, copper, and copper–steel) over the whole data collection period of January–November 2024 and single months with Kruskal–Wallis test and Dunn-test, while superscripted values within rows are significantly different ($p < 0.05$); N = 695.

	Steel	N	Copper	N	Copper–Steel	N	*p*-Value
All months	29.5 (13.8–37) [a]	611	31.9 (17–36.8) [a]	58	27.9 (21.4–36.2)	22	a = 0.002
January	21.5 (17–26)	10					
February	21.8 (13.8–27.7) [b, c]	49	26.4 (25.6–29.4) [c]	3	27.7 (26.4–28.8) [b]	4	b = 0.004. c = 0.044
March	23.6 (15.5–32.5) [d]	35	28 (27.4–30.7) [d]	4	24.9 (21.4–26.2)	3	d = 0.013
April	28.7 (18.1–32.6) [e]	63	31.5 (29.7–32.9) [e]	8	30.2 (30.2–30.2)	1	e < 0.001
May	28.9 (24.6–32.8) [f]	66	32.1 (31.3–32.9) [f]	8	30.3 (29.9–30.6)	3	f < 0.001
June	31.7 (24.6–37)	97	33.1 (29.1–36.8)	12	33.4 (33.4–33.4)	1	
July	33.6 (29.1–36.2)	50	34 (31.3–36.4)	4	31.1 (31.1–31.1)	1	
August	33 (28.2–36.1)	77	34.5 (29.8–36)	6	33 (32.2–36.2)	4	
September	32.9 (23.1–36.6)	70	33 (29.7–34.1)	5			
October	21.9 (15.5–25.1)	46	22.2 (17–25.6)	6	24.2 (22.5–26.6)	3	
November	21.9 (14.9–32.4)	48	25.7 (24–27.7)	3	24.2 (22.4–26)	2	

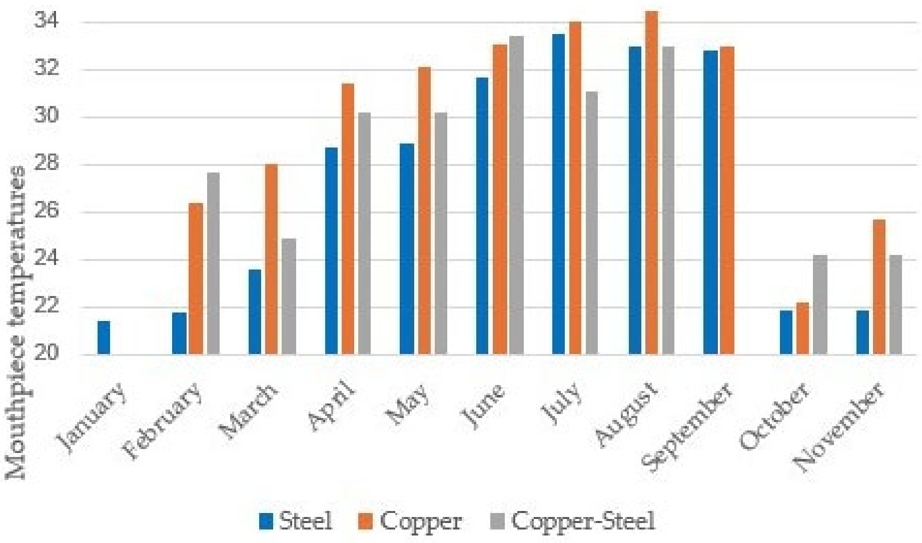

Figure S1. Mouthpiece temperatures of different bit materials (steel, copper, and copper–steel) over the single months. Temperatures are in °C.

Table S4. Median values (min–max) of mouthpiece temperature [°C] between bit shapes (Butterfly Liverpool, Liverpool bit, and Loose Ring Snaffle with 4 rings) over the whole data collection period of January-November 2024 and single months with Kruskal–Wallis test and Dunn-test, while superscripted values within rows are significantly different ($p < 0.05$); N = 692.

	Butterfly Liverpool	N	Liverpool Bit	N	Loose Ring Snaffle with 4 Rings	N	*p*-Value
All months	29 (13.8–36.6) [a]	415	28.9 (15.5–37)	120	31.5 (14.9–36.8) [a]	157	a < 0.001
January	21.6 (17–26)	9	20.5 (20.5–20.5)	1			
February	21.8 (13.8–29.4)	43	23.9 (15.5–28.1)	13			
March	23.7 (16–30.7)	30	25.4 (15.5–32.5)	10	26.7 (24.9–28.5)	2	
April	28.7 (22.5–32.4) [b]	42	28.3 (26.4–30.5) [c]	13	30.8 (18.1–32.9) [b, c]	17	b = 0.001. c = 0.01
May	29.1 (25–32.6)	49	28.9 (24.7–32.8)	13	31.3 (24.6–32.9)	15	

June	31.7 (25.3–36.3)	58	32.8 (28.2–37)	20	33.1 (29.8–36)	32
July	33.3 (29.8–36.2)	33	33.7 (29.1–36.4)	6	33.5 (30.6–35.9)	16
August	33 (30.1–35.9)	47	34.2 (28.2–36.2)	17	33 (29.8–36)	23
September	31.4 (23.1–36.6)	42	33.3 (23.3–36.6)	11	33.2 (23.4–34.5)	22
October	21.6 (15.5–25.1)	31	23.1 (17.3–26.6)	8	22.2 (17–25.6)	16
November	22 (17.8–32.4)	31	22.6 (17.4–26)	8	22.8 (14.9–27.7)	14

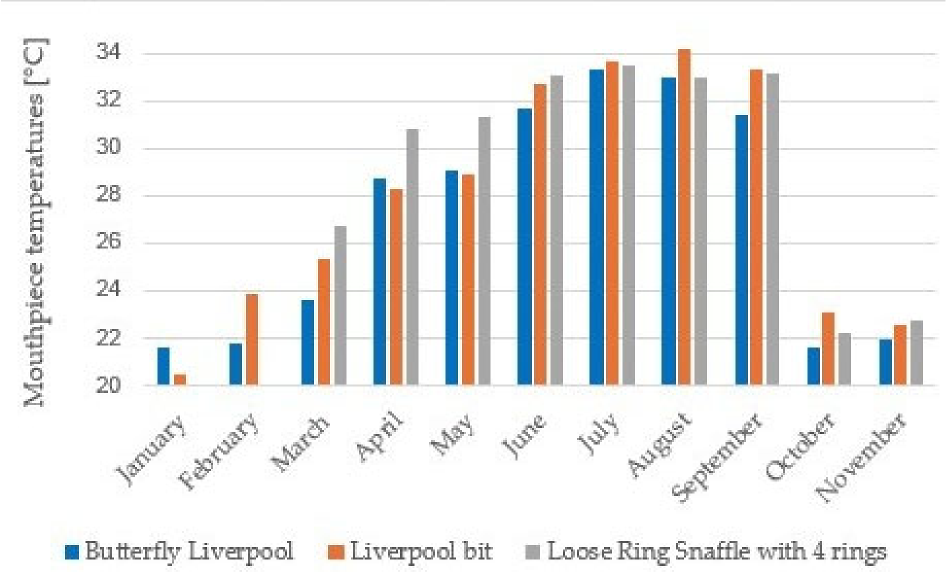

Figure S2. Mouthpiece temperatures of different bit shapes over the months; median bit temperatures [°C] of the exposed part of the mouthpieces of different bit shapes (Butterfly Liverpool, Liverpool, and Loose Ring Snaffle with 4 rings) over 11 months.

Table S5. Median values (min–max) of mouthpiece temperatures [°C] of the bit configurations in the groups of WBGTs. Four groups of WBGTs (<10°, 10–20°, 20–25°, and >25°) were formed to document differences between bit configurations using Friedman's test followed by Conover's post hoc test. Post hoc significance values were adjusted using the Bonferroni correction for multiple tests. Superscript a indicates a significant difference between the Liverpool bit steel and the Liverpool bit copper–steel in the category WBGT > 25 °C ($p < 0.026$).

Bit Shape	WBGT < 10 °C	WBGT 10–20 °C	WBGT 20–25 °C	WBGT > 25 °C
Butterfly Liverpool steel	21.4 (13.8–29.4) N = 58	25.3 (15.5–35.5) N = 172	31.4 (25.3–36.6) N = 97	33.85 (29.3–36.3) N = 88
Liverpool bit steel	23 (15.5–26.5) N = 13	26 (17.3–32.5) N = 45	31.45 (28.2–36.6) N = 24	34.2 (30.2–37.0) [a] N = 25
Liverpool bit copper–steel	26.2 (26–28.1) N = 3	28.15 (24–30.2) N= 4	32.6 (31.1–36.4) N = 3	35.3 (33.4–36.2) [a] N = 3
Loose Ring Snaffle with 4 rings steel	17.65 (14.9–23.4) N = 6	23.7 (18.1–31.3) N = 29	31.3 (24.6–34.5) N = 37	33.95 (30.6–36.3) N = 32
Loose Ring Snaffle with 4 rings copper–steel	22.4 N = 1	25.9 (22.5–30.6) N = 4	32.8 (32.2–33.4) N = 3	-
Loose Ring Snaffle with 4 rings copper	-	29.7 (17–32.9) N= 19	32.75 (29.8–34.9) N = 20	34.8 (31.3–36.8) N = 8
N	81	273	183	156

Table S6. Median values (min–max) of mouthpiece temperatures [°C] for the bit configurations (defined by shape and material) over the whole data collection period (all months) and the single months.

	Butterfly Liverpool Steel	Liverpool Bit Steel	Liverpool Bit Copper–Steel	Loose Ring Snaffle with 4 Rings Steel	Loose Ring Snaffle with 4 Rings Copper–Steel	Loose Ring Snaffle with 4 Rings Copper
All months	29<br>(13.8–36.6)<br>N = 415	28.7<br>(15.5–37)<br>N = 107	30.2<br>(24–36.4)<br>N = 13	31.2<br>(14.9–36.3)<br>N = 103	27.6<br>(22.4–33.4)<br>N = 7	32.4<br>(36.8–17)<br>N = 47
January	21.6<br>(17–26)<br>N = 9	20.5<br>(20.5–20.5)<br>N = 1				
February	21.8<br>(13.8–29.4)<br>N = 43	23.6<br>(15.5–20.5)<br>N = 12	28.1<br>N = 1			
March	23.7<br>(16–30.7)<br>N = 30	24.7<br>(15.5–32.5)<br>N = 9	26.2<br>N = 1	24.9<br>N = 1		28.5<br>N = 1
April	28.7<br>(22.5–32.4)<br>N = 42	28.3<br>(26.4–30.5)<br>N = 12	30.2<br>N = 1	29.7<br>(18.1–32.6)<br>N = 10		31.3<br>(39.7–32.9)<br>N = 7
May	29.1<br>(25–32.6)<br>N = 49	28.9<br>(24.7–32.8)<br>N = 13		26<br>(24.6–30)<br>N = 5	29.1<br>(27.6–30.6)<br>N = 2	32.1<br>(31.3–32.9)<br>N = 8
June	31.7<br>(25.3–36.2)<br>N = 58	32.7<br>(28.3–37)<br>N = 19	33.4<br>(31.25–33.4)<br>N = 2	31.25<br>(24.6–36.3)<br>N = 20		33.1<br>(29.1–36.8)<br>N = 12
July	33.3<br>(29.8–36.2)<br>N = 33	33.7<br>(29.1–35.9)<br>N = 4	33.75<br>(31.1–36.4)<br>N = 2	33.9<br>(30.6–35.9)<br>N = 13		33.1<br>(31.3–34.9)<br>N = 3
August	33<br>(30.1–35.9)<br>N = 47	34.2<br>(28.2–36.1)<br>N = 14	35.3<br>(32.6–35.3)<br>N = 2	32.8<br>(30.4–35.3)<br>N = 16	32.8<br>(32.2–33.4)<br>N = 2	34.3<br>(29.9–36)<br>N = 5
September	31.4<br>(23.1–36.6)<br>N = 42	33.3<br>(23.3–36.6)<br>N = 10	29.7<br>N = 1	33.2<br>(23.4–34.5)<br>N = 18		33.1<br>(32.4–34.1)<br>N = 4
October	21.6<br>(15.5–25.1)<br>N = 31	22.8<br>(15.5–25)<br>N = 7	26.6<br>N = 1	22.2<br>(20.9–23.6)<br>N = 9	23.4<br>(22.5–24.2)<br>N = 2	22.2<br>(17–25.6)<br>N = 5
November	22<br>(17.8–32.4)<br>N = 31	21.6<br>(17.4–24.2)<br>N = 6	25<br>(24–26)<br>N = 2	20.8<br>(14.9–24.3)<br>N = 11	22.4<br>N = 1	26.7<br>(25.7–27.7)<br>N = 2
